# Oxymatrinium tetra­chloridoferrate(III)

**DOI:** 10.1107/S1600536812000281

**Published:** 2012-01-11

**Authors:** Xiong He, Xing Chuan Wei, Yu Chang Tian, Jia Xiong Lai

**Affiliations:** a School of Chemistry and Chemical Engineering, Guangzhou University, Guangzhou 510000, People’s Republic of China

## Abstract

The asymmetric unit of the title compound, (C_15_H_25_N_2_O_2_)[FeCl_4_], contains a tetra­chloridoferrate(III) anion and a oxymatrinium cation [oxymatrine is (4*R*,7a*S*,13a*R*,13b*R*,13c*S*)-dodeca­hydro-1*H*,5*H*,10*H*-dipyrido[2,1-*f*:3′,2′,1′-*ij*][1,6]naphthyridin-10-one 4-oxide]. The conformation of oxymatrine is similar to that of matrine with one ring having a half-chair conformation, while the others have chair conformations. Chiral chains of cations along the *c* axis are formed by O—H⋯O hydrogen bonds.

## Related literature

For related structures, see: Chen *et al.* (2011 ▶); Jin *et al.* (2005 ▶, 2009 ▶); Zhang *et al.* (2003 ▶). For hydrogen-bond motifs, see: Bernstein *et al.* (1995 ▶). For the biological activity of oxymatrine, see: Song *et al.* (2006 ▶); Wang *et al.* (2005 ▶); Xiang *et al.* (2002 ▶); Zhang *et al.* (2001 ▶, 2009 ▶); Sun *et al.* (2008 ▶). Oxymatrine is an alkaloid extracted from the Chinese herb *Sophora alopecuraides* L, see: Lai *et al.* (2003 ▶). For the preparation and studies of related salts, see: Mao *et al.* (2008 ▶); Li (2006 ▶).
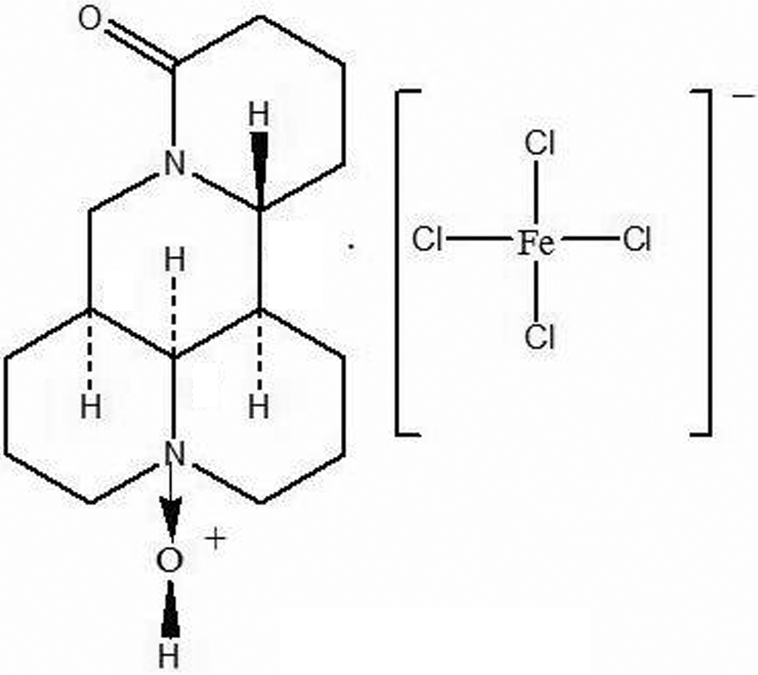


## Experimental

### 

#### Crystal data

(C_15_H_25_N_2_O_2_)[FeCl_4_]
*M* *_r_* = 463.02
Orthorhombic, 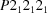

*a* = 7.7919 (4) Å
*b* = 11.9518 (6) Å
*c* = 21.1315 (10) Å
*V* = 1967.92 (17) Å^3^
*Z* = 4
Mo *K*α radiation
μ = 1.32 mm^−1^
*T* = 173 K
0.45 × 0.26 × 0.25 mm

#### Data collection

Bruker SMART 1000 CCD diffractometer
Absorption correction: multi-scan (*SADABS*; Sheldrick, 2004 ▶) *T*_min_ = 0.588, *T*_max_ = 0.734
9963 measured reflections
4267 independent reflections
3812 reflections with *I* > 2σ(*I*)
*R*_int_ = 0.020

#### Refinement

*R*[*F* ^2^ > 2σ(*F* ^2^)] = 0.026
*wR*(*F* ^2^) = 0.061
*S* = 1.03
4267 reflections
218 parameters
H-atom parameters constrained
Δρ_max_ = 0.31 e Å^−3^
Δρ_min_ = −0.28 e Å^−3^
Absolute structure: Flack (1983 ▶), 1787 Friedel pairs
Flack parameter: 0.006 (14)

### 

Data collection: *SMART* (Bruker, 2001 ▶); cell refinement: *SAINT-Plus* (Bruker, 2003 ▶); data reduction: *SAINT-Plus*; program(s) used to solve structure: *SHELXTL* (Sheldrick, 2008) ▶; program(s) used to refine structure: *SHELXTL*; molecular graphics: *SHELXTL*; software used to prepare material for publication: *SHELXTL*.

## Supplementary Material

Crystal structure: contains datablock(s) I, global. DOI: 10.1107/S1600536812000281/mw2043sup1.cif

Structure factors: contains datablock(s) I. DOI: 10.1107/S1600536812000281/mw2043Isup2.hkl

Additional supplementary materials:  crystallographic information; 3D view; checkCIF report

## Figures and Tables

**Table 1:** Hydrogen-bond geometry (Å, °)

*D*—H⋯*A*	*D*—H	H⋯*A*	*D*⋯*A*	*D*—H⋯*A*
O2—H2⋯O1^i^	0.84	1.76	2.5935 (19)	171

Symmetry code: (i) 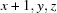.

**Fig. 1.**
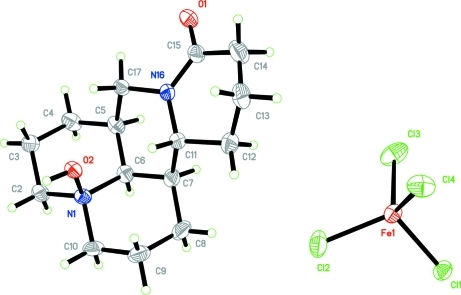
The asymmetric unit of (I) with atom labels and 50% probability displacement ellipsoids.

**Fig. 2.**
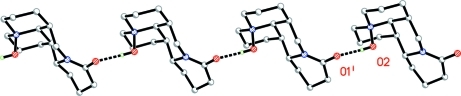
Part of the packing of (I) showing the chiral chain running along the c axis. Hydrogen bonds are depicted as dashed lines. H atoms not involved in these interactions have been omitted.

## supplementary crystallographic information

### Comment

Oxymatrine is an alkaloid extracted from the Chinese herb Sophora alopecuraides *L* (Lai *et al.*, 2003). It has been reported that oxymatrine plays important roles as an anti-arrhythmic, in immunity regulation, as an anti-tumor agent among other applications (Song *et al.*, 2006). It is extensively used in China for treatment of viral hepatitis, cancer, cardiac diseases (such as viral myocarditis), and skin diseases (such as psoriasis and eczema) (Wang *et al.*, 2005). A mechanistic study showed that oxymatrine could inhibit apoptotic cell death in hepatocytes (Xiang *et al.*, 2002) as well as scavenge hydroxyradicals and influence ion channels of cardiomyocytes (Sun *et al.*, 2008). The synthesis of similar compounds has been reported (Jin *et al.*, 2005).

The asymmetric unit of (I) is illustrated in Fig. 1. The geometry of the [FeCl_4_]^-^ ion compares favorably with that reported previously (Zhang *et al.*, 2003). In the oxymatrinium cation (oxygen O2 is protonated) (Fig. 1), the D ring (containing atom C15) has a half-chair conformation while the other rings adopt chair forms. The cations are linked *via* O—H···O hydrogen bonds forming a zigzag chain motif (Fig. 2, Table 1).

### Experimental

A mixture of FeCl_3_.6H_2_O (0.135 g, 0.5 mmol) and oxymatrine (0.132 g, 0.5 mmol) dissolved in ethanol (20 ml) was refluxed with stirring. A light-yellow precipitate appeared after a few minutes and an aqueous HCl solution (1 *M*) was added drop-wise until the solution became clear. After standing for two days yellow prismatic crystals were observed which were immediately recovered by filtration and copiously washed with methanol.Yellow single crystals of the title compound suitable for X-ray structure determination were recrystallized from ethanol by slow evaporation of the solvents at room temperature over several days.

### Refinement

All H atoms were placed in calculated positions and allowed ride on their parent atoms at distances of 0.84 Å (O—H), 0.99 Å (methylene) and 1.00 Å (methyne), and constrained to ride on their parent atoms with *U*_iso_(H) = 1.5 times *U*_eq_(O) and 1.2 times *U*_eq_ (C–methylene and C–methyne), respectively.

### Crystal data

**Table:**

(C_15_H_25_N_2_O_2_)[FeCl_4_]	*F*(000) = 956
*M**_r_* = 463.02	*D*_x_ = 1.563 Mg m^−^^3^
Orthorhombic, *P*2_1_2_1_2_1_	Mo *K*α radiation, λ = 0.71073 Å
Hall symbol: P 2ac 2ab	Cell parameters from 6437 reflections
*a* = 7.7919 (4) Å	θ = 2.6–27.1°
*b* = 11.9518 (6) Å	µ = 1.32 mm^−^^1^
*c* = 21.1315 (10) Å	*T* = 173 K
*V* = 1967.92 (17) Å^3^	Prism, yellow
*Z* = 4	0.45 × 0.26 × 0.25 mm

### Data collection

**Table:**

Bruker SMART 1000 CCD diffractometer	4267 independent reflections
Radiation source: fine-focus sealed tube	3812 reflections with *I* > 2σ(*I*)
graphite	*R*_int_ = 0.020
ω scans	θ_max_ = 27.1°, θ_min_ = 1.9°
Absorption correction: multi-scan (*SADABS*; Sheldrick, 2004)	*h* = −9→9
*T*_min_ = 0.588, *T*_max_ = 0.734	*k* = −7→15
9963 measured reflections	*l* = −23→27

### Refinement

**Table:**

Refinement on *F*^2^	Secondary atom site location: difference Fourier map
Least-squares matrix: full	Hydrogen site location: inferred from neighbouring sites
*R*[*F*^2^ > 2σ(*F*^2^)] = 0.026	H-atom parameters constrained
*wR*(*F*^2^) = 0.061	*w* = 1/[σ^2^(*F*_o_^2^) + (0.0334*P*)^2^] where *P* = (*F*_o_^2^ + 2*F*_c_^2^)/3
*S* = 1.03	(Δ/σ)_max_ = 0.001
4267 reflections	Δρ_max_ = 0.31 e Å^−^^3^
218 parameters	Δρ_min_ = −0.28 e Å^−^^3^
0 restraints	Absolute structure: Flack (1983), 1787 Friedel pairs
Primary atom site location: structure-invariant direct methods	Flack parameter: 0.006 (14)

### Special details

**Table:**

Geometry. All e.s.d.'s (except the e.s.d. in the dihedral angle between two l.s. planes) are estimated using the full covariance matrix. The cell e.s.d.'s are taken into account individually in the estimation of e.s.d.'s in distances, angles and torsion angles; correlations between e.s.d.'s in cell parameters are only used when they are defined by crystal symmetry. An approximate (isotropic) treatment of cell e.s.d.'s is used for estimating e.s.d.'s involving l.s. planes.
Refinement. Refinement of *F*^2^ against ALL reflections. The weighted *R*-factor *wR* and goodness of fit *S* are based on *F*^2^, conventional *R*-factors *R* are based on *F*, with *F* set to zero for negative *F*^2^. The threshold expression of *F*^2^ > σ(*F*^2^) is used only for calculating *R*-factors(gt) *etc*. and is not relevant to the choice of reflections for refinement. *R*-factors based on *F*^2^ are statistically about twice as large as those based on *F*, and *R*- factors based on ALL data will be even larger.

### Fractional atomic coordinates and isotropic or equivalent isotropic displacement parameters (Å^2^)

**Table:**

	*x*	*y*	*z*	*U*_iso_*/*U*_eq_	
Fe1	0.48378 (4)	0.01347 (2)	0.169548 (12)	0.02484 (8)	
Cl1	0.48652 (8)	−0.12257 (4)	0.23956 (2)	0.03028 (12)	
Cl2	0.72310 (9)	0.10944 (6)	0.17647 (3)	0.04546 (17)	
Cl3	0.25496 (9)	0.11672 (6)	0.18583 (3)	0.04688 (18)	
Cl4	0.46744 (8)	−0.05810 (5)	0.07447 (2)	0.03739 (14)	
N1	1.1039 (2)	0.60782 (15)	0.13234 (8)	0.0221 (4)	
C2	1.1716 (3)	0.72196 (19)	0.14985 (10)	0.0276 (5)	
H2A	1.2898	0.7305	0.1334	0.033*	
H2B	1.1759	0.7290	0.1965	0.033*	
C3	1.0591 (3)	0.81319 (19)	0.12295 (11)	0.0298 (5)	
H3A	1.0615	0.8094	0.0762	0.036*	
H3B	1.1048	0.8870	0.1358	0.036*	
C4	0.8751 (3)	0.8018 (2)	0.14585 (11)	0.0325 (5)	
H4A	0.8030	0.8576	0.1237	0.039*	
H4B	0.8708	0.8189	0.1917	0.039*	
C5	0.8001 (3)	0.68571 (19)	0.13492 (10)	0.0259 (5)	
H5	0.6964	0.6806	0.1627	0.031*	
C6	0.9204 (3)	0.59318 (19)	0.15665 (9)	0.0248 (5)	
H6	0.9271	0.5996	0.2038	0.030*	
C7	0.8473 (3)	0.47659 (19)	0.14298 (10)	0.0272 (5)	
H7	0.7408	0.4703	0.1692	0.033*	
C8	0.9697 (3)	0.38652 (19)	0.16806 (11)	0.0381 (5)	
H8A	0.9718	0.3896	0.2149	0.046*	
H8B	0.9267	0.3118	0.1555	0.046*	
C9	1.1505 (3)	0.40203 (19)	0.14288 (12)	0.0366 (6)	
H9A	1.2269	0.3446	0.1615	0.044*	
H9B	1.1506	0.3921	0.0964	0.044*	
C10	1.2176 (3)	0.51723 (19)	0.15907 (10)	0.0298 (5)	
H10A	1.2239	0.5253	0.2056	0.036*	
H10B	1.3351	0.5258	0.1419	0.036*	
C11	0.7893 (3)	0.46034 (17)	0.07363 (9)	0.0235 (4)	
H11	0.8923	0.4642	0.0455	0.028*	
C12	0.7037 (3)	0.34673 (18)	0.06509 (11)	0.0328 (5)	
H12A	0.7916	0.2870	0.0663	0.039*	
H12B	0.6216	0.3335	0.1000	0.039*	
C13	0.6095 (3)	0.3433 (2)	0.00198 (12)	0.0369 (6)	
H13A	0.6897	0.3611	−0.0329	0.044*	
H13B	0.5620	0.2675	−0.0054	0.044*	
C14	0.4664 (3)	0.4280 (2)	0.00382 (12)	0.0402 (6)	
H14A	0.4237	0.4395	−0.0398	0.048*	
H14B	0.3706	0.3968	0.0291	0.048*	
C15	0.5150 (3)	0.53988 (18)	0.03113 (9)	0.0267 (4)	
N16	0.6714 (2)	0.55307 (14)	0.05703 (8)	0.0213 (4)	
C17	0.7358 (3)	0.66798 (17)	0.06706 (10)	0.0232 (4)	
H17A	0.6427	0.7220	0.0580	0.028*	
H17B	0.8308	0.6829	0.0371	0.028*	
O1	0.41292 (18)	0.62004 (14)	0.02719 (7)	0.0319 (4)	
O2	1.09826 (17)	0.59837 (13)	0.06528 (6)	0.0218 (3)	
H2	1.1963	0.6109	0.0503	0.033*	

### Atomic displacement parameters (Å^2^)

**Table:**

	*U*^11^	*U*^22^	*U*^33^	*U*^12^	*U*^13^	*U*^23^
Fe1	0.03331 (16)	0.02190 (14)	0.01931 (13)	−0.00039 (14)	0.00051 (12)	−0.00073 (11)
Cl1	0.0359 (3)	0.0272 (2)	0.0277 (2)	−0.0024 (3)	−0.0005 (2)	0.00547 (19)
Cl2	0.0571 (4)	0.0398 (4)	0.0395 (3)	−0.0238 (3)	−0.0061 (3)	0.0081 (3)
Cl3	0.0607 (4)	0.0372 (4)	0.0427 (4)	0.0213 (3)	0.0135 (3)	0.0028 (3)
Cl4	0.0444 (3)	0.0461 (3)	0.0216 (2)	0.0090 (3)	−0.0043 (2)	−0.0082 (2)
N1	0.0239 (8)	0.0255 (9)	0.0169 (8)	−0.0008 (8)	−0.0052 (7)	−0.0010 (8)
C2	0.0260 (11)	0.0290 (12)	0.0277 (11)	−0.0047 (9)	−0.0072 (9)	−0.0038 (10)
C3	0.0318 (12)	0.0205 (10)	0.0372 (12)	−0.0016 (9)	−0.0087 (9)	−0.0025 (9)
C4	0.0284 (11)	0.0307 (13)	0.0383 (13)	0.0028 (10)	−0.0043 (10)	−0.0100 (11)
C5	0.0211 (10)	0.0325 (13)	0.0240 (11)	0.0007 (9)	0.0035 (8)	−0.0054 (9)
C6	0.0249 (10)	0.0335 (12)	0.0160 (10)	−0.0037 (9)	0.0030 (8)	−0.0001 (9)
C7	0.0281 (10)	0.0297 (12)	0.0237 (10)	−0.0040 (10)	0.0018 (8)	0.0046 (10)
C8	0.0486 (14)	0.0297 (11)	0.0359 (12)	−0.0076 (11)	−0.0129 (12)	0.0132 (10)
C9	0.0415 (13)	0.0247 (12)	0.0436 (14)	0.0044 (10)	−0.0142 (11)	0.0045 (11)
C10	0.0322 (11)	0.0299 (12)	0.0272 (11)	0.0048 (10)	−0.0105 (9)	0.0030 (10)
C11	0.0203 (9)	0.0239 (11)	0.0265 (11)	−0.0011 (8)	0.0002 (8)	0.0028 (9)
C12	0.0337 (12)	0.0228 (11)	0.0419 (13)	−0.0032 (10)	−0.0013 (10)	0.0007 (10)
C13	0.0362 (13)	0.0304 (13)	0.0441 (14)	−0.0097 (11)	0.0005 (11)	−0.0084 (11)
C14	0.0318 (13)	0.0401 (13)	0.0487 (14)	−0.0093 (12)	−0.0080 (11)	−0.0059 (11)
C15	0.0210 (10)	0.0336 (11)	0.0257 (10)	−0.0028 (10)	0.0029 (9)	0.0030 (8)
N16	0.0190 (8)	0.0225 (9)	0.0226 (9)	−0.0022 (7)	0.0014 (6)	0.0005 (7)
C17	0.0204 (10)	0.0214 (10)	0.0278 (10)	0.0004 (8)	−0.0010 (9)	−0.0013 (9)
O1	0.0194 (7)	0.0359 (9)	0.0404 (9)	−0.0004 (7)	−0.0015 (6)	0.0040 (8)
O2	0.0190 (7)	0.0307 (8)	0.0157 (6)	−0.0011 (6)	0.0001 (5)	−0.0008 (6)

### Geometric parameters (Å, °)

**Table:**

Fe1—Cl4	2.1874 (6)	C8—H8A	0.9900
Fe1—Cl2	2.1942 (7)	C8—H8B	0.9900
Fe1—Cl3	2.1954 (7)	C9—C10	1.512 (3)
Fe1—Cl1	2.1984 (5)	C9—H9A	0.9900
N1—O2	1.422 (2)	C9—H9B	0.9900
N1—C2	1.509 (3)	C10—H10A	0.9900
N1—C10	1.509 (3)	C10—H10B	0.9900
N1—C6	1.529 (3)	C11—N16	1.482 (3)
C2—C3	1.510 (3)	C11—C12	1.523 (3)
C2—H2A	0.9900	C11—H11	1.0000
C2—H2B	0.9900	C12—C13	1.523 (3)
C3—C4	1.520 (3)	C12—H12A	0.9900
C3—H3A	0.9900	C12—H12B	0.9900
C3—H3B	0.9900	C13—C14	1.507 (3)
C4—C5	1.523 (3)	C13—H13A	0.9900
C4—H4A	0.9900	C13—H13B	0.9900
C4—H4B	0.9900	C14—C15	1.505 (3)
C5—C6	1.521 (3)	C14—H14A	0.9900
C5—C17	1.534 (3)	C14—H14B	0.9900
C5—H5	1.0000	C15—O1	1.248 (3)
C6—C7	1.533 (3)	C15—N16	1.345 (3)
C6—H6	1.0000	N16—C17	1.478 (3)
C7—C8	1.533 (3)	C17—H17A	0.9900
C7—C11	1.546 (3)	C17—H17B	0.9900
C7—H7	1.0000	O2—H2	0.8400
C8—C9	1.518 (4)		
			
Cl4—Fe1—Cl2	108.37 (3)	C7—C8—H8B	109.3
Cl4—Fe1—Cl3	108.45 (3)	H8A—C8—H8B	107.9
Cl2—Fe1—Cl3	112.70 (3)	C10—C9—C8	110.7 (2)
Cl4—Fe1—Cl1	109.23 (2)	C10—C9—H9A	109.5
Cl2—Fe1—Cl1	109.48 (3)	C8—C9—H9A	109.5
Cl3—Fe1—Cl1	108.55 (3)	C10—C9—H9B	109.5
O2—N1—C2	109.09 (15)	C8—C9—H9B	109.5
O2—N1—C10	109.52 (15)	H9A—C9—H9B	108.1
C2—N1—C10	110.59 (15)	N1—C10—C9	111.44 (17)
O2—N1—C6	107.26 (14)	N1—C10—H10A	109.3
C2—N1—C6	110.36 (17)	C9—C10—H10A	109.3
C10—N1—C6	109.95 (16)	N1—C10—H10B	109.3
N1—C2—C3	110.95 (16)	C9—C10—H10B	109.3
N1—C2—H2A	109.4	H10A—C10—H10B	108.0
C3—C2—H2A	109.4	N16—C11—C12	111.55 (17)
N1—C2—H2B	109.4	N16—C11—C7	108.17 (16)
C3—C2—H2B	109.4	C12—C11—C7	110.63 (18)
H2A—C2—H2B	108.0	N16—C11—H11	108.8
C2—C3—C4	111.3 (2)	C12—C11—H11	108.8
C2—C3—H3A	109.4	C7—C11—H11	108.8
C4—C3—H3A	109.4	C13—C12—C11	109.82 (18)
C2—C3—H3B	109.4	C13—C12—H12A	109.7
C4—C3—H3B	109.4	C11—C12—H12A	109.7
H3A—C3—H3B	108.0	C13—C12—H12B	109.7
C3—C4—C5	113.30 (19)	C11—C12—H12B	109.7
C3—C4—H4A	108.9	H12A—C12—H12B	108.2
C5—C4—H4A	108.9	C14—C13—C12	108.42 (19)
C3—C4—H4B	108.9	C14—C13—H13A	110.0
C5—C4—H4B	108.9	C12—C13—H13A	110.0
H4A—C4—H4B	107.7	C14—C13—H13B	110.0
C6—C5—C4	112.33 (17)	C12—C13—H13B	110.0
C6—C5—C17	112.53 (17)	H13A—C13—H13B	108.4
C4—C5—C17	113.14 (19)	C15—C14—C13	114.89 (19)
C6—C5—H5	106.0	C15—C14—H14A	108.5
C4—C5—H5	106.0	C13—C14—H14A	108.5
C17—C5—H5	106.0	C15—C14—H14B	108.5
C5—C6—N1	113.07 (17)	C13—C14—H14B	108.5
C5—C6—C7	112.02 (17)	H14A—C14—H14B	107.5
N1—C6—C7	112.84 (18)	O1—C15—N16	120.96 (19)
C5—C6—H6	106.1	O1—C15—C14	119.75 (19)
N1—C6—H6	106.1	N16—C15—C14	119.22 (19)
C7—C6—H6	106.1	C15—N16—C17	118.37 (17)
C8—C7—C6	110.00 (17)	C15—N16—C11	124.78 (17)
C8—C7—C11	114.93 (19)	C17—N16—C11	116.77 (15)
C6—C7—C11	113.68 (17)	N16—C17—C5	111.93 (17)
C8—C7—H7	105.8	N16—C17—H17A	109.2
C6—C7—H7	105.8	C5—C17—H17A	109.2
C11—C7—H7	105.8	N16—C17—H17B	109.2
C9—C8—C7	111.76 (18)	C5—C17—H17B	109.2
C9—C8—H8A	109.3	H17A—C17—H17B	107.9
C7—C8—H8A	109.3	N1—O2—H2	109.5
C9—C8—H8B	109.3		
			
O2—N1—C2—C3	59.1 (2)	C2—N1—C10—C9	179.15 (19)
C10—N1—C2—C3	179.66 (18)	C6—N1—C10—C9	57.0 (2)
C6—N1—C2—C3	−58.5 (2)	C8—C9—C10—N1	−58.5 (2)
N1—C2—C3—C4	57.9 (2)	C8—C7—C11—N16	179.53 (16)
C2—C3—C4—C5	−52.4 (3)	C6—C7—C11—N16	−52.5 (2)
C3—C4—C5—C6	47.8 (2)	C8—C7—C11—C12	57.1 (2)
C3—C4—C5—C17	−81.0 (2)	C6—C7—C11—C12	−174.92 (18)
C4—C5—C6—N1	−48.8 (2)	N16—C11—C12—C13	45.7 (2)
C17—C5—C6—N1	80.3 (2)	C7—C11—C12—C13	166.21 (18)
C4—C5—C6—C7	−177.63 (17)	C11—C12—C13—C14	−64.4 (2)
C17—C5—C6—C7	−48.6 (2)	C12—C13—C14—C15	44.5 (3)
O2—N1—C6—C5	−64.5 (2)	C13—C14—C15—O1	170.62 (19)
C2—N1—C6—C5	54.2 (2)	C13—C14—C15—N16	−6.5 (3)
C10—N1—C6—C5	176.50 (16)	O1—C15—N16—C17	−14.0 (3)
O2—N1—C6—C7	63.9 (2)	C14—C15—N16—C17	163.0 (2)
C2—N1—C6—C7	−177.34 (16)	O1—C15—N16—C11	169.15 (18)
C10—N1—C6—C7	−55.1 (2)	C14—C15—N16—C11	−13.8 (3)
C5—C6—C7—C8	−177.77 (18)	C12—C11—N16—C15	−6.8 (3)
N1—C6—C7—C8	53.3 (2)	C7—C11—N16—C15	−128.66 (19)
C5—C6—C7—C11	51.7 (2)	C12—C11—N16—C17	176.39 (17)
N1—C6—C7—C11	−77.2 (2)	C7—C11—N16—C17	54.5 (2)
C6—C7—C8—C9	−53.7 (2)	C15—N16—C17—C5	129.11 (19)
C11—C7—C8—C9	76.1 (2)	C11—N16—C17—C5	−53.8 (2)
C7—C8—C9—C10	56.7 (2)	C6—C5—C17—N16	48.7 (2)
O2—N1—C10—C9	−60.6 (2)	C4—C5—C17—N16	177.31 (17)

### Hydrogen-bond geometry (Å, °)

**Table:**

*D*—H···*A*	*D*—H	H···*A*	*D*···*A*	*D*—H···*A*
O2—H2···O1^i^	0.84	1.76	2.5935 (19)	171

Symmetry codes: (i) *x*+1, *y*, *z*.
